# Perioperative Risk and Modifiable Targets in Sequential Pulmonary Resection Within One Year

**DOI:** 10.3390/jcm15187252

**Published:** 2026-09-18

**Authors:** Jae M. Cho, Fatemehsadat Pezeshkian, Justin S. Heidel, José A. Rodriguez Zamboni, Michael T. Jaklitsch, Hassan A. Khalil

**Affiliations:** 1 Division of Thoracic Surgery, Department of Surgery, Mass General Brigham, Boston, MA 02115, USA; jmcho@bwh.harvard.edu (J.M.C.); yasaman.pezeshkian@yahoo.com (F.P.);; 2 Department of Surgery, Harvard Medical School, Boston, MA 02115, USA

**Keywords:** sequential pulmonary resection, completion lobectomy, ipsilateral reoperation, prolonged air leak, perioperative outcomes, within-patient paired analysis

## Abstract

**Background**: Sequential pulmonary resections are heterogeneous clinical scenarios that may increase perioperative risks for patients. This study uses within-patient paired comparisons to characterize the perioperative experience of sequential operations, including optimal timing to return, impact of operative sequence, and complication rates. **Methods**: A single-institution retrospective review was conducted of 231 patients who underwent sequential pulmonary resections within one year between 2017 and 2025. Paired analysis was conducted for perioperative outcomes at the first (S1) and second (S2) operations. The primary outcome was major morbidity (Clavien–Dindo ≥ IIIa) at S2. A multivariable logistic regression model was fit for major morbidity at S2 with pre-specified predictors. Firth penalized logistic regression was performed with sensitivity analysis. **Results**: Across the entire cohort, major morbidity was similar at S1 and S2 (5% to 7%, *p* = 0.503). Sequential lobectomy+ (OR 4.87, 95% CI 1.02–23.20, *p* = 0.047) and ipsilateral re-entry (OR 4.48, 95% CI 1.31–15.36, *p* = 0.017) were both associated with major morbidity. Both associations persisted under Firth penalization (OR 4.99, 95% CI 1.02–20.54 and OR 4.06, 95% CI 1.32–14.34). No specific time interval was associated with major morbidity, whether modeled categorically or continuously. Prolonged air leak was the only complication that rose significantly at S2 (4% to 10%, *p* = 0.011), and was associated with increased length of stay after S2. **Conclusions**: Major morbidity did not increase significantly at S2 for sequential pulmonary resections within one year, and no interval within the year was shown to be safer than another. As the magnitude of the surgery (sequential lobectomy) and ipsilateral re-entry were most strongly associated with risk independent of timing, those patients should be counseled appropriately. Prolonged air leak prevention is a potentially modifiable target for enhanced recovery pathways adapted to reoperative patients.

## 1. Introduction

Sequential pulmonary resections represent an increasingly common challenge in thoracic surgery [1,2]. Indications for reoperation within one year are heterogeneous and include completion lobectomy after sublobar resection, staged bilateral resection for synchronous multifocal disease, or reoperations for new primary/metastatic lung cancer [3,4]. Each of these scenarios has been well studied in its own corner of the literature. Completion lobectomy has primarily been studied for its oncologic outcomes, while staged bilateral resection has compared one-stage versus two-stage approach in propensity-matched cohorts [5,6]. The technical challenges of ipsilateral re-entry have also been described, attributed to intrathoracic adhesions (up to 80% of cases), leading to elevated rates of conversion to open [7,8]. However, the risk of the second operation within one year has not been addressed as a unified problem.

Patients undergoing a second pulmonary resection within one year may have ongoing physiologic recovery and reduced pulmonary reserve [2]. Understanding the risks associated with the second pulmonary resection is important for operative planning, patient counseling, and even the decision to proceed. Identifying factors associated with increased morbidity can inform operative planning at both the index and second operations.

In this study, we conduct a within-patient paired analysis of sequential pulmonary resection performed within one year at a single high-volume center. Our objective is to analyze anatomic and timing factors that predict reoperative risk, and to explore differences in postoperative complications.

## 2. Patients and Methods

### 2.1. Patients and Cohort

This study was approved by the Brigham and Women’s Hospital Institutional Review Board (Protocol 2024P001400, approval 4 June 2024), and informed consent was waived.

**Figure 1:**
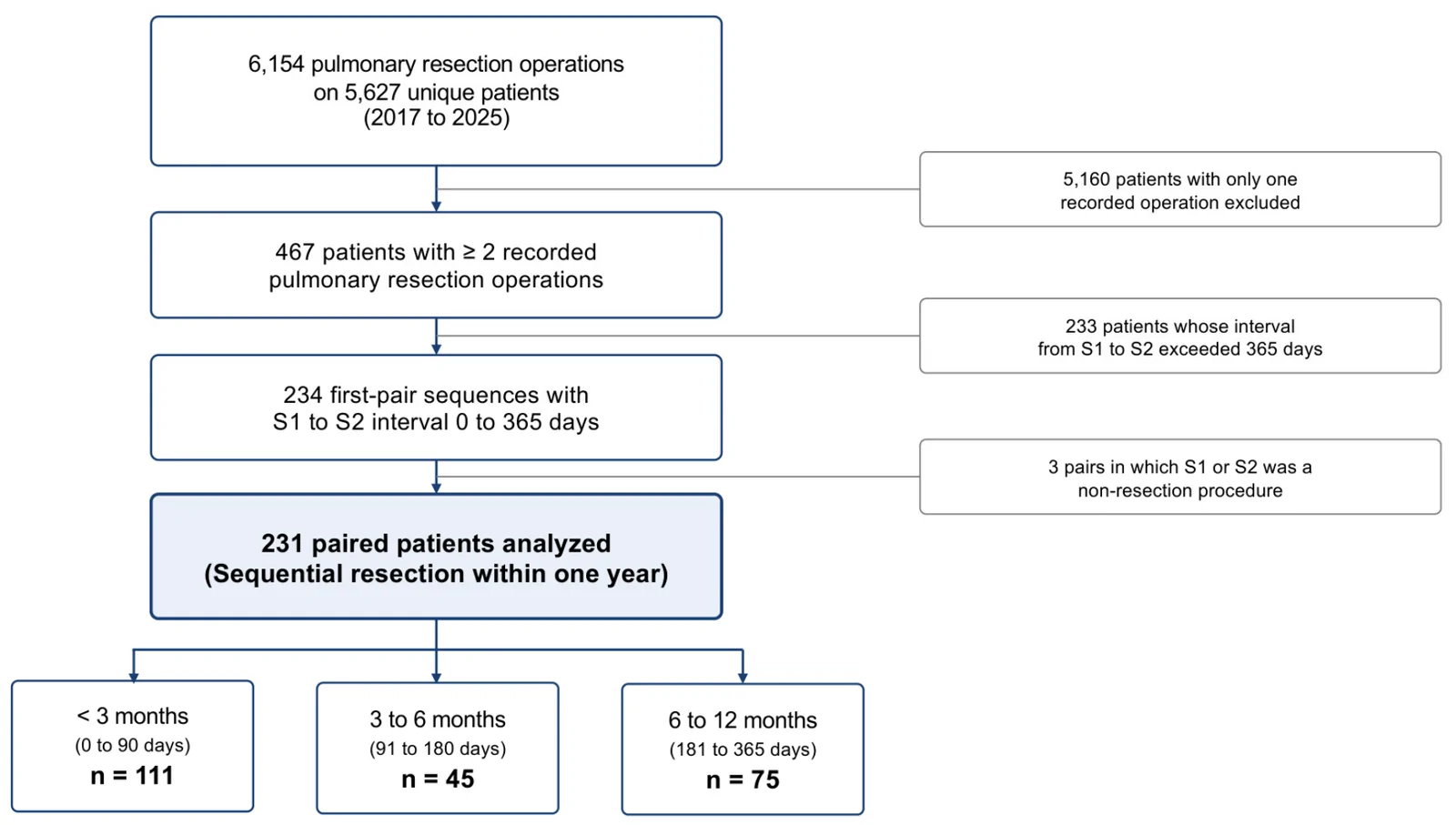
Cohort flow. The 231-pair analytic cohort selected from the 5627-patient source database, with downstream split across the three pre-specified interval bins.

We queried the institutional thoracic surgery database for all pulmonary resection operations performed at Brigham and Women’s Hospital from 2017 through 2025. Out of 6154 operations for 5627 patients, patients who underwent sequential pulmonary resections in one year were extracted. Inclusion criteria included patients who (1) underwent at least two recorded operations, with the two events recorded as surgery 1 (S1) and surgery 2 (S2), (2) had an interval between S1 and S2 of less than or equal to 365 days, and (3) had both operations classified as a resection (wedge resection, segmentectomy, lobectomy, or pneumonectomy). Under these criteria, the analytic cohort was made up of 231 paired patients. The study cohort flow diagram is shown in Figure 1. Patient characteristics were collected from retrospective chart review of the electronic medical record and included demographics, operative details, and postoperative hospital course. Indication was characterized using the documented surgical intent and final pathology at each operation. Operative approach was recorded as the initial intended approach, with conversions to open thoracotomy marked separately.

### 2.2. Variables and Outcome Definitions

Procedure type was assigned to each operation with the hierarchy pneumonectomy > lobectomy > segmentectomy > wedge resection. Procedure-type pairs were assigned as Wedge → Wedge, Wedge → Lobe+, Lobe+ → Wedge, Segmentectomy → Lobe+, Lobe+ → Lobe+, or other patterns comprising any remaining combination. Lobe+ refers to a combined cohort of lobectomy and pneumonectomy, due to the low number of pneumonectomies completed in this timeframe. Because lobectomy and pneumonectomy differ substantially in extent and risk, a sensitivity analysis excluding the two pneumonectomies is reported. However, the outlined procedure sequence above is reported for discussion. Laterality was also defined for each operation and labeled as right or left. An ipsilateral pair is defined as two operations in which the side at S1 and the side at S2 were the same.

The interval was stratified into three groups: less than 3 months (0 to 90 days), 3 to 6 months (91 to 180 days), and 6 to 12 months (181 to 365 days). The intervals were further modeled as a continuous variable.

The primary outcome was major morbidity, defined as any complication with Clavien–Dindo grade IIIa or higher at the second operation [9]. Any complication included grade 2 or higher. Prolonged air leak was defined as air leak persisting more than five days. In-hospital, 30-day, and 90-day mortality are reported as secondary outcomes. Because we did not pre-specify a non-inferiority margin, the study does not aim to formally establish non-inferiority of sequential resections.

### 2.3. Statistical Analysis

Nonparametric continuous data are presented as median and interquartile range and compared using the Wilcoxon signed-rank test for paired samples or the Mann–Whitney U test for independent samples. Categorical data are presented as *n* (%) and compared using McNemar’s exact test for paired binary outcomes, Fisher’s exact test for independent groups, or the Cochran–Armitage trend test across the three ordered interval timeframe groups. The pre-specified primary outcome was major morbidity (Clavien–Dindo ≥ IIIa) at S2. Missing data were handled with no imputation, using complete-case analysis throughout.

A multivariable logistic regression model was conducted with pre-specified predictors (anatomic resection pattern, ipsilateral re-entry, interval between operations). Calendar era was added to the model as a sensitivity analysis. Odds ratios were reported with Wald 95% confidence intervals and Wald *p*-values. The model was refitted using Firth penalized regression with penalized profile-likelihood confidence intervals and penalized likelihood-ratio *p*-values. A two-sided *p*-value <0.05 was considered statistically significant. All analyses were conducted, and figures were created in R (R version 4.4.3, R Foundation for Statistical Computing, Vienna, Austria) using RStudio (version 2026.04.0+526, Posit Software, Boston, MA, USA). The ggplot package was used for the creation of figures [10].

## 3. Results

### 3.1. Cohort

Of 5627 patients, 231 underwent sequential pulmonary resection operations within 365 days and met all inclusion criteria. Mean age at S1 was 62.1 ± 13.1 years, 132 (57.1%) were female, and median Charlson Comorbidity Index was 3 (IQR 2–6). Of 173 pairs with recorded smoking history, 35.3% were never smokers, 53.8% were former smokers, and 11.0% were current smokers. The median interval from S1 to S2 was 91 days (IQR 44–226). 111 (48.1%) patients had reoperation in less than 3 months, 45 (19.5%) between 3 and 6 months, and 75 (32.5%) between 6 and 12 months. Full baseline characteristics and demographics are presented in Table 1. FEV1 was recorded for 163 patients (71%) before S1 and 138 (60%) before S2, with a median of 90% and 88% predicted respectively. Surgical intent at S2 was curative in 184/198 with fields recorded. Final pathology of the resected lesion at S2 was malignant in 180 of 224.

### 3.2. Paired Perioperative Outcomes

In paired comparison of S1 versus S2 for all patients (Table 2), major morbidity was similar between S1 and S2 (4.8%, 95% CI 2.4–8.4, to 6.5%, 95% CI 3.7–10.5; McNemar *p* = 0.503). Any complication (16.5% to 20.8%, *p* = 0.237), return to OR (2.6% to 1.7%), and 30-day readmission were also similar between S1 and S2 (4.8% to 3.9%). In-hospital mortality rose from 0 to 4, although not statistically significant (*p* = 0.125). The low number of events provides limited power to exclude a clinically meaningful difference. The median length of stay was equal at 2 days at both operations but shifted at the upper interquartile range from 1–3 days to 1–4 days (*p* < 0.001). 30-day mortality was 2.2% (95% CI 0.6–5.4) after S2, and 90-day mortality was 2.8% (95% CI 0.9–6.4). After censoring follow-up at the date of S2, there were no mortalities within 30 or 90 days after S1; 90-day mortality after S2 was 4 of 14 in evaluable Lobe+ → Lobe+ pairs (28.6%, 95% CI 8.4–58.1) compared with 1 of 166 in all other pairs (0.6%, 95% CI 0.0–3.3). Individual complications causing major morbidity after S2 included: pleural effusion (3), pneumothorax (3), prolonged air leak (3), excessive secretions (2), hemothorax (2), respiratory failure/insufficiency (2), PEA arrest (2), hemoptysis (1), and renal failure (1). Some patients had more than one complication.

### 3.3. Predictors of Morbidity

**Figure 2:**
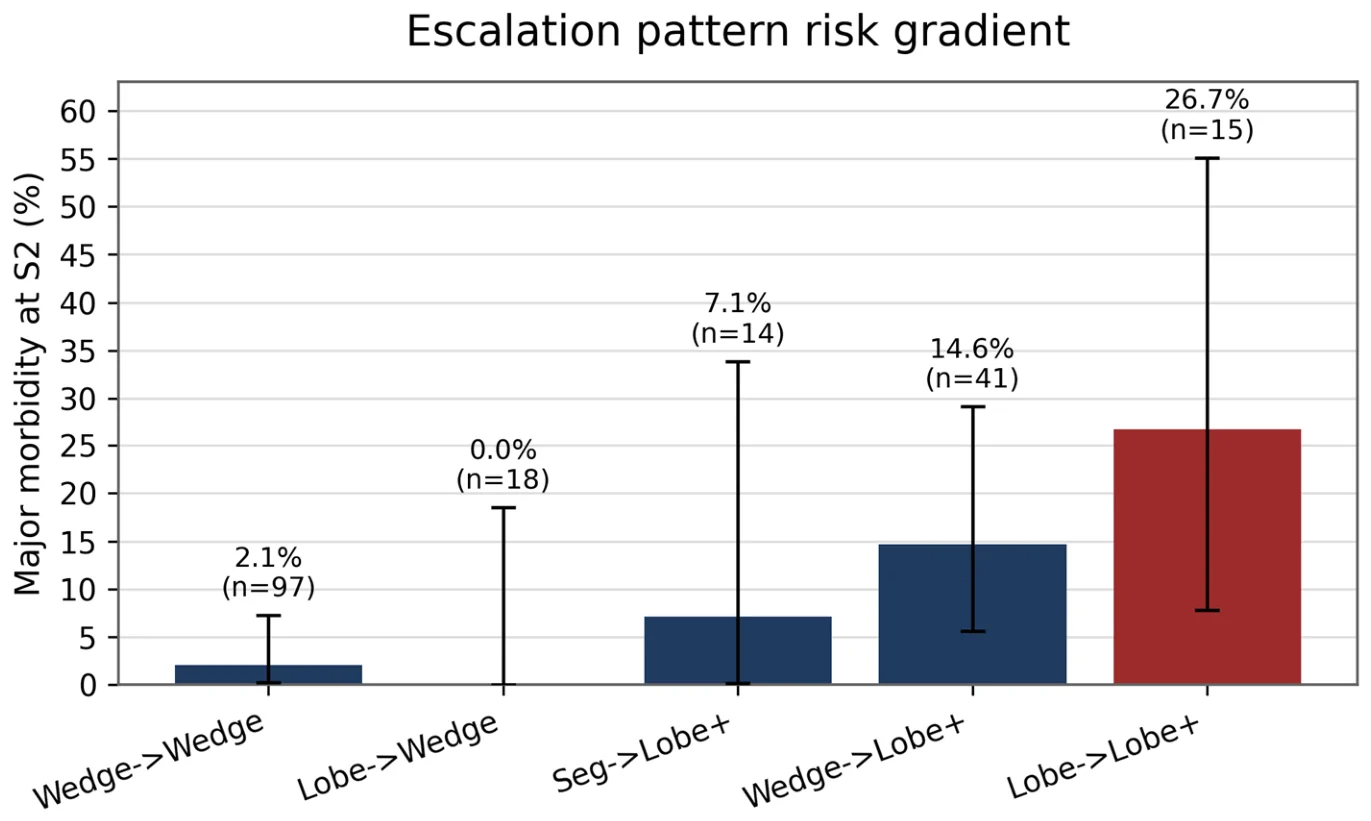
Operative-anatomy risk gradient. Major morbidity at the second operation across the five-bin escalation pattern. The Lobe+ → Lobe+ pattern is highlighted as the high-risk archetype, and 95% confidence intervals are shown around each proportion.

**Figure 3:**
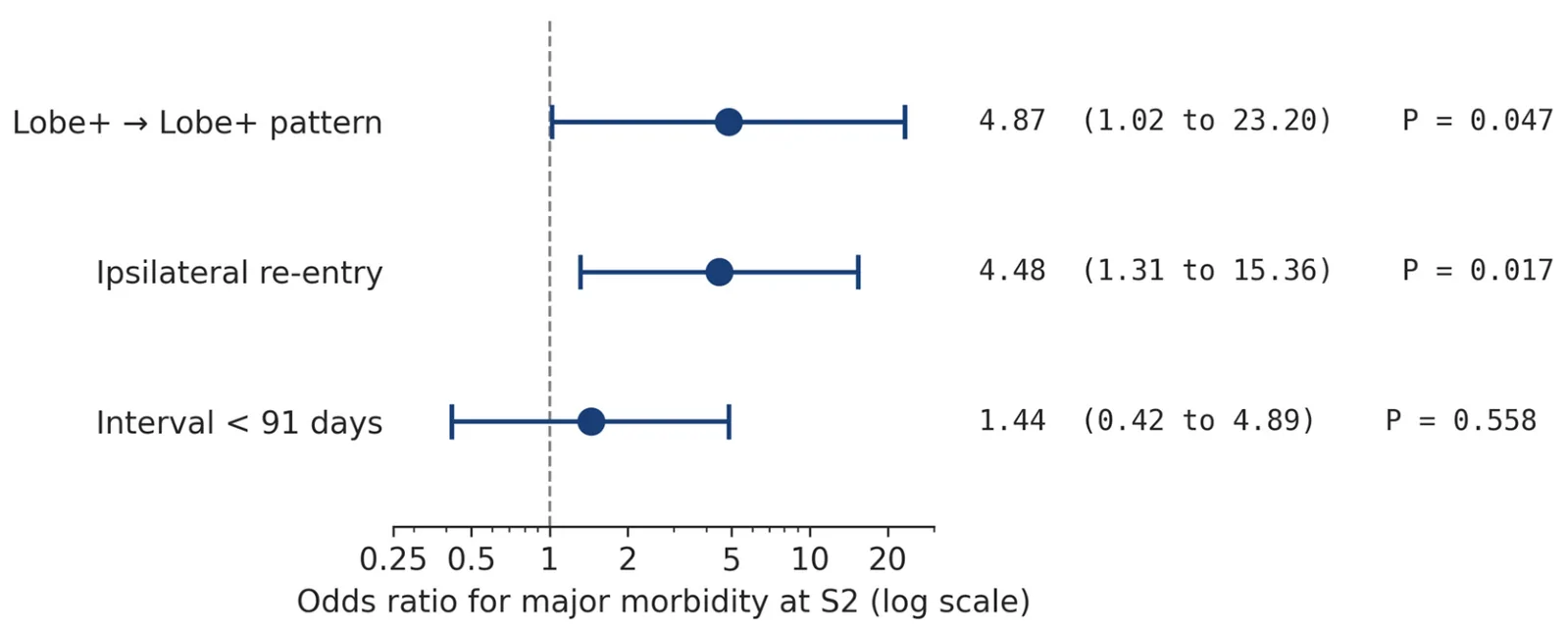
Multivariable logistic regression for major morbidity at the second operation. Forest plot of three pre-specified binary predictors with odds ratios and 95% confidence intervals on a log scale; dashed reference line at OR = 1. *n* = 226 paired patients, 13 events.

Figure 2 illustrates the unadjusted gradient of major morbidity at S2 across all anatomic resection patterns. Major morbidity at S2 increases from 2.1% in Wedge → Wedge to 26.7% in Lobe+ → Lobe+. After multivariable logistic regression for major morbidity at S2, the Lobe+ → Lobe+ escalation pattern had an odds ratio of 4.87 (95% CI 1.02–23.20, *p* = 0.047), and ipsilateral re-entry had an odds ratio of 4.48 (95% CI 1.31–15.36, *p* = 0.017) (Table 3, Figure 3). The three predefined time intervals did not differ meaningfully on any outcome (Table 4), and no timeframe emerged as safer than the others. An interval from S1 to S2 of less than 3 months was not associated with major morbidity (OR 1.44, 95% CI 0.42–4.89, *p* = 0.558). Under reanalysis with Firth penalization, the estimates remain similar with Lobe+ → Lobe+ OR 4.99 (95% CI 1.02–20.54, *p* = 0.048), ipsilateral re-entry OR 4.06 (95% CI 1.32–14.34, *p* = 0.014), and interval under 91 days OR 1.39 (95% CI 0.43–4.71, *p* = 0.578). Modeling the interval continuously resulted in an OR of 1.03 per additional 30 days (95% CI 0.87–1.21, *p* = 0.679). Because the Lobe+ → Lobe+ pattern clustered in the earliest returns, the model was refitted excluding those pairs. Major morbidity remained unassociated with interval (OR 1.05 per 30 days, 95% CI 0.88–1.24, *p* = 0.586) while ipsilateral re-entry remained associated (OR 4.57, 95% CI 1.31–19.27, *p* = 0.017). Adding calendar era to our model again did not change the estimates (era OR 1.24, 95% CI 0.38–4.53, *p* = 0.723), although robotic use did increase from 4.2% in 2017–2020 to 20.0% in 2021–2025 (*p* < 0.001).

For the 15 Lobe+ → Lobe+ pairs, perioperative outcomes at S2 were globally worse than in the remaining 216 pairs across every endpoint (Table 5). Major morbidity at S2 was 26.7% vs. 5.1% (*p* = 0.010), and any complication was 60.0% vs. 18.1% (*p* = 0.001). In-hospital mortality was 26.7% versus 0% (four deaths in 15 cases, *p* < 0.001). The operation was more often performed open via thoracotomy (46.7% vs. 12.6%, *p* < 0.001), was longer (median 159 vs. 102 min, *p* = 0.001), and the median length of stay was 5 vs. 2 days (*p* = 0.002).

There were two pneumonectomies in the lobectomy+ group, both performed as completion pneumonectomy at S2. These were the only operations involving pneumonectomy in the overall cohort. Excluding these two cases, the pure Lobe → Lobe pairs (*n* = 13) held the same pattern as the Lobe+ analysis compared to the remainder of the cohort. Major morbidity was 23.1% (*p* = 0.036), any complication was 61.5% (*p* = 0.001), in-hospital mortality was 23.1% (3 deaths, *p* < 0.001), and open approach was 41.7% (*p* = 0.020). The median operative time was 159 min (*p* = 0.001), and the median length of stay was 6 days (*p* = 0.003).

All four in-hospital deaths followed a Lobe+ → Lobe+ pattern, and three of the four were urgent or emergent reoperations for a complication related to the index operation. The three indications were as follows: (1) completion pneumonectomy 60 days after right lobectomy for a pulmonary artery-to-bronchus fistula, resulting in death from PEA arrest on postoperative day 3; (2) reoperation for right middle lobe torsion and obstruction after right lobectomy, resulting in death from respiratory failure on postoperative day 6; and (3) reoperation for refractory hydropneumothorax and empyema from bronchopleural fistula after right lobectomy, resulting in death from respiratory failure on postoperative day 7. The fourth operation was elective for contralateral completion lobectomy for metastatic disease, resulting in death from PEA arrest of undetermined cause on postoperative day 7. None of the four patients received interval adjuvant therapy between operations, and patients elected comfort measures only prior to death in all four.

Among the 226 pairs with recorded laterality, 77 (34.1%) were ipsilateral reoperations. Compared with contralateral pairs, ipsilateral reoperation was associated with significantly worse perioperative outcomes at S2 (Table 6). Major morbidity was 11.7% vs. 2.7% (*p* = 0.012), any complication was 31.2% vs. 14.8% (*p* = 0.005), and completion with an open or thoracotomy approach was 28.4% versus 6.0% (*p* < 0.001). Operative time nearly doubled (median 151 vs. 83 min, *p* < 0.001) and length of stay extended by a median of 2 days (4 vs. 2, *p* < 0.001). In-hospital mortality and 30-day readmission were numerically higher in the ipsilateral group but did not reach statistical significance.

### 3.4. Prolonged Air Leak and Increased Length of Stay

Prolonged air leak was the only individual complication that rose significantly from S1 to S2 (3.9% to 9.5%, *p* = 0.011). For the 22 patients whose second operation was complicated by prolonged air leak, the median length of stay was 9 days, compared to 2 days in the pairs without air leak at S2. After excluding the 22 patients with air leak at S2, the length of stay *p*-value increased to 0.017 from <0.001, but remained statistically significant. This exclusion sensitivity analysis does not establish that air leak causes excess length of stay, and prolonged air leaks themselves are related to the extent of resection, baseline emphysema, and technical factors.

## 4. Discussion

In our within-patient paired analysis of 231 sequential pulmonary resections within one year, two key anatomical factors, sequential lobectomy+ and ipsilateral re-entry, were associated with major morbidity at the second operation. Timing, on the other hand, was not associated with major morbidity. Paired major morbidity did not rise significantly at the second operation, but the absence of a significant difference does not equate to equivalence. Prolonged air leak was the single complication that rose significantly after S2, which may contribute to the increased length of stay after S2. To our knowledge, this study represents the largest within-patient paired analysis of perioperative outcomes after sequential pulmonary resection within one year.

We expand upon prior within-patient paired analysis of postoperative complications after repeat pulmonary resections. Motono et al. studied 41 patients undergoing repeat resection for lung cancer at a single Japanese center and reported similar complication rates between the first and second operations (29% vs. 29%), with no significant differences between ipsilateral and contralateral cases [11]. However, their cohort was limited to oncologic indications, grouped all intervals into a single cohort, and was not adequately powered for multivariable adjustment. Our analysis extends the paired observation to a larger cohort while restricting to a one-year window in which the patient is likely still physiologically recovering from the index operation. We also adjust for laterality and escalation patterns as pre-specified predictors. Our finding that major morbidity did not differ significantly between sequential operations aligns with Motono et al.’s findings, but we additionally report the operative anatomy gradient that emerges from the adjusted model.

Our findings also warrant comparison with a recent contralateral resection analysis by Kadomatsu et al., who reported on 123 patients undergoing contralateral repeat pulmonary resections at a single institution [12]. They found that a surgical interval of three months was associated with 30-day postoperative complications. However, we find that there was no independent association between timing and major morbidity at the second operation. Several methodological differences likely explain these discrepancies. Our cohort was made up of both contralateral and ipsilateral pairs, and our analysis was adjusted for the anatomic escalation pattern, particularly the Lobe+ → Lobe+ pattern, which carries much of the complication risk that an interval-only analysis would capture indirectly. The apparent short-interval effect may reflect the distribution of high-magnitude operations clustering in the early returns rather than an independent interval mechanism. In our data, all four in-hospital deaths at S2 fell within the <3-month interval, and all four occurred in Lobe+ → Lobe+ pairs, supporting this interpretation.

Reddington et al. recently reported a national-database propensity-score matched analysis of oncologic sequential pulmonary resection versus single resections, finding overall comparable safety and increasing stepwise risk with increasing resection magnitude [2]. They also found that ipsilateral sequential resections had longer operative times with greater complication rates, although it was not associated with worsened 30-day composite morbidity and mortality. The presented analysis is complementary to the findings by Reddington et al., while providing a within-patient paired design and limiting analysis to a 1-year temporal window that the national database cannot support. Forster et al. similarly reported short-term outcomes after repeated anatomical resection for a second primary lung cancer, finding the approach feasible and safe [13].

The overall findings from our analysis are intuitive and consistent with non-paired analyses in the literature. The Lobe+ → Lobe+ pattern is the maximally escalating sequence and carried both the highest morbidity and mortality risk. Ipsilateral re-entry doubled operative time, increased the rate of open thoracotomy or conversion to open, resulting in a median two-day longer hospital stay. These findings are consistent with previous reports of severe adhesions leading to additional difficulty of ipsilateral reoperations [7]. Across all examined complications, prolonged air leak was the only complication that rose significantly between operations, and may contribute to the second-operation length of stay increase. The association between prolonged air leak and increased length of hospital stay has been previously identified in the literature, and addressing prolonged air leak is a potentially modifiable target for perioperative quality improvement in enhanced recovery pathways for sequential resections [14,15]. Prolonged air leaks are influenced by resection extent, emphysema, and technical factors, and whether these interventions, such as buttressing of staple lines, application of sealants, or revised drainage protocols, translate to a clinical difference is not tested here and is a direction for prospective work [16,17].

The negative finding on interval timing is also important for patient counseling. Across time intervals (both categorically or continuously), major morbidity, complications, length of stay, and operative time were similar. Patient counseling to accelerate or defer the second operation on the basis of interval alone is not supported. Rather, counseling should address the operative anatomy and indication. If no timing within the year carries increased independent risk, the interval between operations is not a delay to be minimized, but rather a window for prehabilitation before a second operation, especially for those slow to recover from the first. However, these results should be interpreted cautiously, as no independent association does not equate to equivalence. Randomized evidence supports multimodal prehabilitation before lung resection in high-risk patients, although the optimal protocol is still not defined [18].

There are several limitations to address. First, this is a single-institution retrospective review at a high-volume center, and findings require further validation for generalizability. Second, given the limited sample size and that the events-per-variable in the multivariable model is approximately 4.3, findings should be interpreted as exploratory. The Firth penalized models are reported to address this. Third, we were not able to appropriately characterize complications after pneumonectomy. The cohort contained only two pneumonectomies, both performed at S2 as completion pneumonectomy after a prior lobectomy; pneumonectomies were therefore grouped with lobectomies as Lobe+ rather than analyzed separately. Fourth, as indication was not entered in the model, no claim of independence from indication can be made. Further, objective cardiopulmonary fitness was not available for incorporation in many patients, so physiological reserves could not be modeled. Fifth, the within-patient design controls for time-invariant characteristics only, and factors that change between operations, such as interval therapy, performance status, and recovery from the previous operation, are not controlled.

## 5. Conclusions

Major morbidity did not rise significantly after the second resection during sequential pulmonary resection. No independent association between time intervals and major morbidity was found. Instead, sequential lobectomies and ipsilateral re-entry were most strongly associated with risk at the second operation. Prolonged air leak was significantly increased after the second operation and is a potentially modifiable target for perioperative quality improvement. Future work should extend the framework to a multi-institutional cohort, and test whether targeted air leak prevention attenuates complication risk and length of stay differences.

## Figures and Tables

**Table 1:** Cohort and baseline characteristics.

Variable	Value
Total cohort, paired patients	231
Age at S1, years, mean ± SD	62.1 ± 13.1
Female sex	132 (57.1%)
BMI, kg/m^2^, mean ± SD	27.7 ± 5.8
Charlson Comorbidity Index, median (IQR)	3 (2 to 6)
Smoking status (available *n* = 173)	
Never smoker	61 (35.3%)
Past smoker (stopped >1 month before surgery)	93 (53.8%)
Current smoker (within 30 days of surgery)	19 (11.0%)
Hypertension (available *n* = 173)	85 (49.1%)
Coronary artery disease (available *n* = 171)	24 (14.0%)
History of COPD (available *n* = 173)	21 (12.1%)
Diabetes mellitus (available *n* = 163)	23 (14.1%)
Interval from S1 to S2, days, median (IQR)	91 (44 to 226)
<3 months (0 to 90 days)	111 (48.1%)
3 to 6 months (91 to 180 days)	45 (19.5%)
6 to 12 months (181 to 365 days)	75 (32.5%)
Intended Operative Approach at S1	
Open thoracotomy	5 (2.2%)
VATS	194 (83.9%)
Robotic	32 (13.9%)
Conversion to open	11 (4.8%)
Intended Operative Approach at S2	
Open thoracotomy	13 (5.7%)
VATS	185 (80.0%)
Robotic	31 (13.5%)
Conversion to open	21 (9.1%)
Escalation pattern at S2 vs. S1	
Wedge → Wedge	97 (42.0%)
Wedge → Lobe+	41 (17.7%)
Lobe+ → Wedge	18 (7.8%)
Lobe+ → Lobe+ (sequential lobectomy or greater)	15 (6.5%)
Segmentectomy → Lobe+	14 (6.1%)
Other pattern	46 (19.9%)
Laterality (available *n* = 226)	
Contralateral re-entry	149 (65.9%)
Ipsilateral re-entry	77 (34.1%)

S1, first operation; S2, second operation; SD, standard deviation; IQR, interquartile range; BMI, body mass index; COPD, chronic obstructive pulmonary disease. Available *n* is reported for variables with missing data; complete-case handling is used throughout.

**Table 2:** Paired perioperative outcomes, S1 versus S2.

Outcome	S1	S2	*p*-Value
Major morbidity (Clavien–Dindo ≥ IIIa)	11 (4.8%)	15 (6.5%)	0.503
Any complication (CD ≥ 2)	38 (16.5%)	48 (20.8%)	0.237
Prolonged air leak (>5 days)	9 (3.9%)	22 (9.5%)	0.011
Return to OR	6 (2.6%)	4 (1.7%)	0.754
30-day readmission	11 (4.8%)	9 (3.9%)	0.824
In-hospital mortality	0 (0.0%)	4 (1.7%)	0.125
Hospital length of stay, days, median (IQR)	2 (1–3)	2 (1–4)	<0.001
Operative time, minutes, median (IQR)	95 (52–161)	106 (60–168)	0.177

*p*-values: McNemar’s exact test for binary outcomes; Wilcoxon signed-rank test for continuous outcomes (length of stay, operative time). CD, Clavien–Dindo grade; OR, operating room.

**Table 3:** Multivariable logistic regression for major morbidity at S2.

Predictor	Odds Ratio	95% Confidence Interval	*p*-Value
Lobe+ → Lobe+ escalation pattern (vs. all other patterns)	4.87	1.02 to 23.20	0.047
Ipsilateral re-entry (vs. contralateral)	4.48	1.31 to 15.36	0.017
Interval from S1 to S2 < 91 days (vs. ≥ 91 days)	1.44	0.42 to 4.89	0.558

*p*-values are from the Wald test.

**Table 4:** Outcomes at S2 by interval bin.

Outcome at S2	<3 months (*n* = 111)	3 to 6 months (*n* = 45)	6 to 12 months (*n* = 75)	*p*-Value
Major morbidity	9 (8.1%)	2 (4.4%)	4 (5.3%)	0.421
Any complication	24 (21.6%)	6 (13.3%)	18 (24.0%)	0.786
Prolonged air leak	11 (9.9%)	2 (4.4%)	9 (12.0%)	0.717
Return to OR	1 (0.9%)	1 (2.2%)	2 (2.7%)	0.354
30-day readmission	2 (1.8%)	3 (6.7%)	4 (5.3%)	0.190
In-hospital mortality	4 (3.6%)	0 (0.0%)	0 (0.0%)	0.054
LOS, days, median (IQR)	2 (1–5)	2 (2–4)	2 (1–4)	0.261
Operative time, min, median (IQR)	109 (57–177)	115 (78–177)	96 (60–141)	0.305
Lobe+ → Lobe+ pairs	11 (9.9%)	3 (6.7%)	1 (1.3%)	—

*p*-values: Cochran–Armitage trend test across the three ordered interval bins for binary outcomes; Kruskal–Wallis omnibus test for continuous outcomes. The final row shows the descriptive distribution of the Lobe+ → Lobe+ escalation pattern across intervals.

**Table 5:** Outcome profile, Lobe+ → Lobe+ versus all other patterns.

Outcome at S2	Lobe+ → Lobe+ (*n* = 15)	All Other Patterns (*n* = 216)	*p*-Value
Major morbidity (CD ≥ IIIa)	4 (26.7%)	11 (5.1%)	0.010
Any complication (CD ≥ 2)	9 (60.0%)	39 (18.1%)	0.001
In-hospital mortality	4 (26.7%)	0 (0.0%)	<0.001
Completed open/thoracotomy	7 (46.7%)	27 (12.6%)	<0.001
Prolonged air leak	3 (20.0%)	19 (8.8%)	0.160
Return to OR	1 (6.7%)	3 (1.4%)	0.237
30-day readmission	0 (0.0%)	9 (4.2%)	1.000
Hospital length of stay, days, median (IQR)	5 (4 to 8)	2 (1 to 4)	0.002
Operative time, minutes, median (IQR)	159 (120 to 301)	102 (59 to 160)	0.001

*p*-values: Fisher’s exact test for binary outcomes; Mann–Whitney U test for length of stay and operative time. The Lobe+ group pools lobectomy and pneumonectomy. Two Lobectomy → Pneumonectomy pairs are the only pneumonectomies in the cohort.

**Table 6:** Outcome profile, ipsilateral versus contralateral re-entry.

Outcome at S2	Ipsilateral (*n* = 77)	Contralateral (*n* = 149)	*p*-Value
Major morbidity (CD ≥ IIIa)	9 (11.7%)	4 (2.7%)	0.012
Any complication (CD ≥ 2)	24 (31.2%)	22 (14.8%)	0.005
In-hospital mortality	3 (3.9%)	1 (0.7%)	0.268
Completed open/thoracotomy	21 (28.4%)	9 (6.0%)	<0.001
Prolonged air leak	10 (13.0%)	12 (8.1%)	0.245
Return to OR	2 (2.6%)	1 (0.7%)	0.268
30-day readmission	5 (6.5%)	4 (2.7%)	0.280
Hospital length of stay, days, median (IQR)	4 (2 to 6)	2 (1 to 3)	<0.001
Operative time, minutes, median (IQR)	151 (98 to 211)	83 (50 to 124)	<0.001

Complete-case analysis of laterality (*n* = 226 of 231 pairs). *p*-values: Fisher’s exact for binary outcomes; Mann–Whitney U for length of stay and operative time.

## Data Availability

The de-identified analytic dataset and the reproducible analytic pipeline are available from the corresponding author upon reasonable request.
